# The Role of the Maternal and Child Health Handbook in Developmental Surveillance: The Exploration of Milestone Attainment Trajectories

**DOI:** 10.3389/fpsyt.2022.902158

**Published:** 2022-06-17

**Authors:** Tomoya Hirota, Tomoko Nishimura, Misaki Mikami, Manabu Saito, Kazuhiko Nakamura

**Affiliations:** ^1^Department of Psychiatry and Behavioral Sciences, University of California, San Francisco, San Francisco, CA, United States; ^2^Department of Neuropsychiatry, Graduate School of Medicine, Hirosaki University, Hirosaki, Japan; ^3^Research Centre for Child Mental Development, Hamamatsu University School of Medicine, Hamamatsu, Japan; ^4^Department of Comprehensive Rehabilitation Science, Graduate School of Health Sciences, Hirosaki University, Hirosaki, Japan; ^5^Research Center for Child Mental Development, Graduate School of Medicine, Hirosaki University, Hirosaki, Japan

**Keywords:** developmental surveillance, developmental milestones, milestone attainment, neurodevelopmental disorders, maternal and child health handbook, parent-held child health record

## Abstract

Despite the pivotal role of developmental surveillance in pediatric practice for the early detection of neurodevelopmental disorders (NDDs), there are several barriers, including scarcity of time and staff availability, to its implementation. Additionally, inadequate parental knowledge on what to expect about their child's development contributes to potential delays in the early identification of NDDs. Home-based records (HBRs) are widely used in both high-income and low- and middle-income countries, allowing caregivers to prospectively chart the child's development, including milestone attainment, and thus can be a useful tool for developmental surveillance. Therefore, we analyzed data on milestone attainment from birth to 5 years of age obtained through the home-based records (the Maternal Child Health Handbook: MCHH) in 720 children who attended the Hirosaki Five-year-old children Developmental Health Check-up Study in Hirosaki, Japan to identify trajectory patterns of milestone attainment. Parallel process latent class growth analysis on four milestone domains (motor, social interaction, communication, and self-care) revealed three different trajectories (Class 1: “Consistent milestone attainment” group; 42%, Class 2: “subtle initial delay and catch-up” group; 45%, Class 3: “Consistent failure to attain expected milestones” group; 13%). In Class 3, 90% of children were diagnosed with at least one NDDs at age 5 and approximately 65% of children had autism spectrum disorder and/or intellectual disability, the rate of which was higher than that in the other two classes. Boys and preterm-born children were more likely to be assigned to classes with less favorable trajectories of milestone attainment. Although the use of the MCHH alone does not substitute diagnostic evaluation for NDDs, our study findings suggest the potential utility of the MCHH as a tool to educate parents on what longitudinal patterns of milestone attainment are concerning and require prompt visits to professionals.

## Introduction

Assessment of developmental milestones plays a pivotal role in developmental surveillance (1). Developmental surveillance is an ongoing process of monitoring the status of a child by gathering information about the child's development and behavior from multiple sources, allowing for identifying developmental problems and providing early interventions. Combined with screening through the use of standardized questionnaires, developmental milestones are often reviewed by healthcare professionals in pediatric care. A growing body of research has reported the associations between the child's attainment of developmental milestones (speech, motor, for example) and a range of adult health outcomes (educational attainment, sports participation, for example) (2, 3).

Nevertheless, developmental surveillance is not widespread enough in some regions, mostly in low-income and middle-income countries (LMICs) (4). Additionally, even in some industrialized countries, its penetration is inadequate. For example, a cross-sectional study conducted in the United States reported only 37.1% of children aged 9 through 35 months received developmental surveillance from a health care professional in the year before the survey was conducted (5). Although barriers to the implementation of developmental surveillance exist, including lack of time and staff (6), successful developmental surveillance requires caregivers' adequate understandings of the child's development given their role of bringing their child to professionals. Some caregivers may be unaware of what to look for in their child's early development, missing opportunities for access to timely assessment and subsequent early interventions. From this perspective, providing tools that prospectively assist caregivers in understanding what to expect about their child's developmental milestones can be effective to improve early identification and access to services. For example, the list of developmental milestones provided by the Center for Disease and Control and Prevention (7) may serve as a useful tool for helping parents chart their child's development based on their observation at home. In other countries, parent-held child health records (PHCHRs) in which caregivers can chart the child's development are frequently used for this purpose. Evidence supports the utility of the PHCHRs for educating caregivers in maternal and child health and development (8).

A growing number of countries, both developed and developing countries have implemented the use of the PHCHRs. The PHCHRs comprise two types of records: the Child Personal Health Record, the function of which is restricted to monitoring the child's health and development from birth, and the Maternal and Child Health Handbook (MCHH), which is used to monitor mothers during pregnancy and delivery and postpartum periods and track the child's development (9). The PHCHRs were reported to have positive effects on health-related outcomes and parent knowledge about ante-natal care services and child immunization and nutrition uptakes (9) in LMICs.

In Japan, municipalities distribute the MCHH to all pregnant women under universal health care (10). The MCHH was originally developed in 1947 to reduce infant and maternal mortality rates by providing parents with knowledge about pregnancy and improving early recognition of high-risk pregnancies (11). In addition, parents are expected to monitor maternal health throughout the pregnancy and perinatal periods and chart the child's development, nutrition, and vaccination records. The MCHH and a variety of the PHCHRs are now used in many other countries, including LMICs.

Despite the importance of milestone attainment in child development, no studies within the context of the MCHH have examined the longitudinal patterns of milestone attainment/non-attainment during early childhood and associations between certain attainment patterns and neurodevelopmental outcomes. Data that can be prospectively obtained through the MCHH on the child's developmental milestones can facilitate research assessing its utility in developmental surveillance; however, no such research has been conducted thus far.

Thus, our primary aim of the present study was to identify trajectory patterns of attainment of developmental milestones up to 5 years of age using data from a community sample of children at risk of developmental disabilities in Japan. The results from this study would have implications for the utility of the MCHH and other HBRs as a tool for educating caregivers and healthcare professionals on normative and atypical development in early childhood, facilitating its use in other regions and countries, and fueling global health research. To capture unobserved/latent but distinct profiles of individuals who display similar patterns of developmental trajectories, we employed latent class growth analysis, a person-centered analysis (12). No hypotheses were made for the primary study aim given its exploratory nature. Additionally, we aimed to assess distributional patterns of neurodevelopmental disorder (NDD) diagnoses, including autism spectrum disorder (ASD), attention deficit hyperactivity disorder (ADHD), developmental coordination disorder (DCD), and intellectual disability (ID), of children assigned to each trajectory class. We hypothesized that a large portion of children with NDDs, especially ones with ASD and ID, belong to the trajectory pattern with the least favorable milestone attainment (i.e., failing to attain the expected milestones consistently throughout early childhood) given existing studies reporting early developmental signs in children with certain NDDs (13–15). Lastly, we aimed to examine whether certain trajectory patterns were predicted by the child's sex and the child's gestational age. Based on previous research findings reporting that most developmental milestones were attained at a younger age by girls than boys (16, 17) and that preterm birth was associated with delayed attainment of certain milestones (18, 19), we hypothesized that boys and preterm-born children were more likely to be assigned to less favorable trajectory patterns.

## Methods

This study was approved by the Committee of Medical Ethics of Hirosaki University Graduate School of Medicine. Moreover, the information security policies of the city and committee were followed to protect the personal data of the participants. The primary caregiver provided informed consent for the child's participation in the study.

### Study Setting and Participants

**Figure 1:**
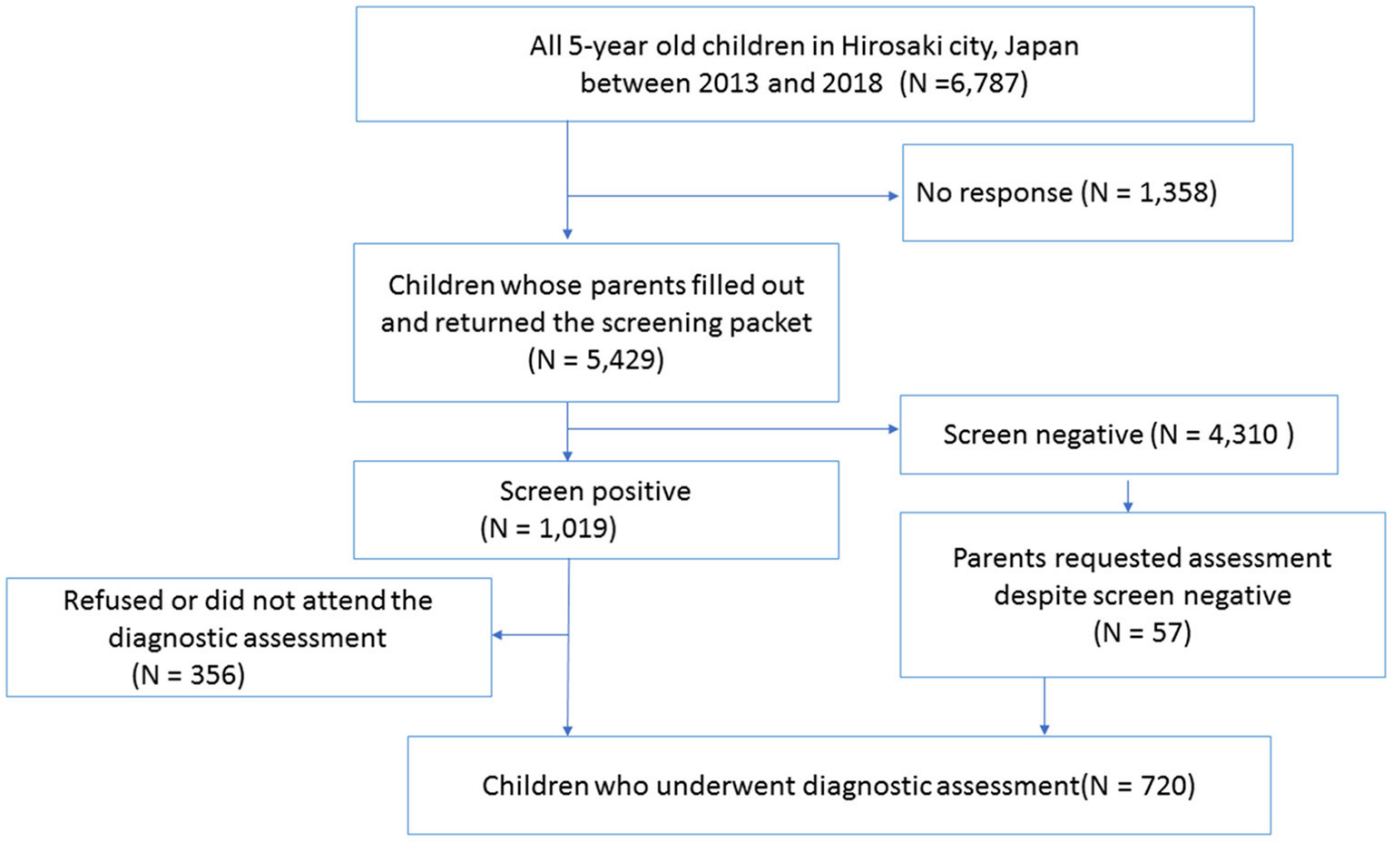
Flow chart of the developmental screening and diagnostic assessment.

This is a secondary data analysis of datasets from the 5-year-old developmental screening and comprehensive assessment conducted between 2013 and 2018 in Hirosaki city, Japan. A total population sample of 5-year-old children residing in this catchment area underwent developmental screening, and those who screened positive were then invited to in-person assessment. At the screening stage, parents of these children filled out questionnaires (see Supplementary Material for details of standardized screening tools used at this stage), which were used to assess the child's development and behaviors. Screen-positive children, defined by our research team (20), were invited to the comprehensive in-person assessment. A sum of 5,429 children underwent the screening, among which 1,019 children were screen-positive. Six hundred and sixty-three out of 1,019 children who screened positive attended the comprehensive assessment. Screen-negative children were also able to attend the assessment if their parents were still concerned about the child's development and requested the assessment (57 children). At the beginning of the assessment, parents of these children provided informed consent to the study, and a total of 720 children attended the in-person assessment (boys 60.3%, mean age at the time of the assessment: 64.6 months with the standard deviation being 2.5 months). At the assessment phase, we conducted child behavior observation and semi-structured parent interviews. Children also underwent cognitive testing and motor skills. Following the assessment stage, a multidisciplinary team, consisting of child and adolescent psychiatrists, a pediatrician, psychologists, occupational therapists reviewed each case and determined the best estimate clinical diagnosis based on the Diagnostic and Statistical Manual of Mental Disorders, Fifth Edition. As such, we ascertained cases for each NDD, including ASD, ID, ADHD, and DCD. Children who received no NDD diagnoses above were categorized as the non-NDD group. Four hundred fifty-five out of 720 children received at least one NDD diagnosis, and the rest of the 265 children were grouped as the non-NDD group. The flow chart of the developmental screening and assessment is represented in Figure 1.

### Measurements and Procedure

#### Milestone Items From the Maternal and Child Health Handbook

The MCHH serves as a record book, where caregivers are expected to chart the child's developmental milestones ranging from 0 to 60 months, vaccination records, and other health information about the child throughout early childhood. In the current study, we extracted the MCHH data from the following data points: between 3 and 4 months, between 6 and 7 months, between 9 and 10 months,12 months, 18 months, 24 months, 36 months, 48 months, and 60 months. For the data analysis, we dichotomized responses for each milestone item to 0 (“My child has not attained this developmental milestone”) and 1 (“My child has already attained this developmental milestone”). Two authors (TH and MS) reviewed each developmental milestone item and assigned them to one of the four developmental domains, including motor, social interaction, communication, and self-help (Table 1).

**Table 1:** Milestone items in the Maternal Child Health Handbook and domains.

**Age**	**Domain**	**Item**
12 months or earlier	Motor	Holds head up (3–4 m)
		Rolls over (6–7 m)
		Sits up without support (6–7 m)
		Reaches for toys nearby (6–7 m)
		Crawls (9–10 m)
		Pulls him/herself up (9–10 m)
		Picks up objects with fingers (9–10 m)
		Walks holding on to things for support (12 m)
	Social interaction	Smile (3–4 m)
		Responds to voice (3–4 m)
		Follows parents (9–10 m)
		Use of gestures (12 m)
	Communication	Babbles (6–7 m)
		Understands simple commands (12 m)
	Self-help	Eats solid food (6–7 m)
		Eats regularly (12 m)
24 months or earlier	Motor	Walks without support (18 m)
		Runs (24 m)
	Social interaction	Responds to the child's name being called (18 m)
		Symbolic play (24 m)
		Imitates adults' behaviors (24 m)
	Communication	Speaks meaningful words (18 m)
		Put two words together (24 m)
	Self-help	Drinks using a cup (18 m)
		Feeds him/herself with a spoon (24 m)
		Eats meats and vegetables (24 m)
36 months or earlier	Motor	Climbs stairs without support (36 m)
		Draws a circle (36 m)
	Social interaction	Pretend play (36 m)
		Has friends (36 m)
	Communication	Knows and says his/her own name (36 m)
	Self-help	Dresses and undresses on his/her own (36 m)
		Brushes teeth and wash hands (36 m)
		Chews foods well (36 m)
48 months or earlier	Motor	Jumps down from steps (48 m)
		Hops on one leg (48 m)
		Draws a cross (48 m)
		Uses scissors (48 m)
	Social interaction	Plays make-believe (48 m)
	Communication	Tells about her/his experiences (48 m)
	Self-help	Dresses and undresses on his/her own (48 m)
		Can urinate alone in a bathroom (48 m)
60 months or earlier	Motor	Does somersault (60 m)
	Social interaction	Enjoys group activities (60 m)
		Shows empathy to others (60 m)
	Communication	Speaks with clear pronunciation (60 m)
	Self-help	Uses a bathroom for bowel movements (60 m)

### Scales and Diagnostic Tools and Testing

At the screening stage, parents filled out questionnaires related to the child's development and their parenting stress using internationally validated scales, including the Autism Spectrum Screening Questionnaire (21), the ADHD Rating Scale (22), the Developmental Coordination Disorder Questionnaire (23), the Strengths and Difficulties Questionnaire (SDQ) (24), and the Parenting Stress Index (25). Screening criteria we set in the developmental checkup can be found elsewhere (20). At the assessment stage, we conducted the Diagnostic Interview for Social Communication Disorders (26), a structured interview with parents used for the diagnosis of ASD and related NDDs. Additionally, children underwent the interview, cognitive testing, and motor functioning assessment [See Supplementary Material 1 and the following reference (20) for details about diagnostic tools and testing].

### Analytic Plans

For data analyses of milestone attainment, we categorized each data point into five different age ranges (12 months or earlier, 24 months or earlier, 36 months or earlier, 48 months or earlier, and 60 months or earlier) (Table 1). We counted the cumulative number of potential delays in each developmental domain. A potential delay was considered if parents reported “My child has not achieved this developmental milestone” in the MCHH.

To identify groups of children who had similar trajectories of milestone attainment, we used parallel process latent class growth analysis, in which the four milestone domains (motor, social interaction, communication, and self-help) were processed concurrently (27). Participants were assigned to the latent classes based on the most likely posterior probabilities. In the process of data analysis, we treated the number of unattained milestones as negative binomial distribution given that the majority of children were expected to attain milestones at the time being reported by their parents in the MCHH.

Class enumeration started with a one-class solution, followed by an exploration of additional models with more latent classes (two-class model, then three-class model, for example). We used the following fit indices: the Akaike information criterion (AIC) (28), Bayesian information criterion (BIC) (29), and Vuong-Lo-Mendell-Rubin likelihood ratio test (VLMR-LRT) (30). Entropy, an indicator of model quality, was also reported; entropy with values approaching 1 indicates a clear delineation of classes (31). To ensure that the models converged on global rather than local solutions, 500 random sets of starting values and 50 final stage optimizations were used. Theoretical justification and interpretability were also considered in determining the optimal number of classes. Following the confirmation of the best fitting class model, we reported proportions of cases with NDD diagnoses (and ones without NDD) per each class.

We then performed multinomial logistic regression analyses with the child's sex and gestational as the independent variables to investigate the influence of these factors on poor prognosis patterns of milestone attainment. Odds ratios (OR) and 95% confidence intervals (CI) were calculated to show the risks of increasing probabilities of assignment to each class when the class representing consistent milestone attainment was taken as the reference class.

Missing data were handled using full information maximum likelihood estimation. Missing data at each time range are summarized in Supplementary Material 2. All analyses were conducted using Mplus version 8.6 (Muthén and Muthén, Los Angeles, CA, USA) and SPSS version 27.0 (IBM Corporation, Armonk, NY, USA).

## Results

The parallel LCGA performed in 720 children at the risk of neurodevelopmental disorders revealed the information to assess the fit of the model (Table 2). After carefully comparing fit indices (AIC, BIC, and *p*-values of BLRT and VLMR-LRT) and taking theoretical justification into account for each class solution, we determined that the three-class solution was the optimal model (please see Supplementary Material 3 for further explanations about the class determination processes).

**Table 2:** Model fit indices and criteria for one- through five-class models.

**N of classes**	**AIC**	**BIC**	**Adjusted BIC**	**VLMR**	**LMR-LRT**	**Entropy**
1	21,578	21,724	21,622	-	-	-
2	18,623	18,828	18,686	<0.0001	<0.0001	0.93
3	17,481	17,745	17,561	<0.0001	<0.0004	0.94
4	16,585	16,908	16,683	0.06	0.06	0.94
5	16,116	16,499	16,232	0.241	0.241	0.94

*AIC, Akaike information criterion; BIC, Bayesian information criterion; VLMR, Vuong–Lo–Mendell–Rubin test p-value; LMR-LRT, Lo–Mendell–Rubin likelihood ratio test p-value*.

**Figure 2:**
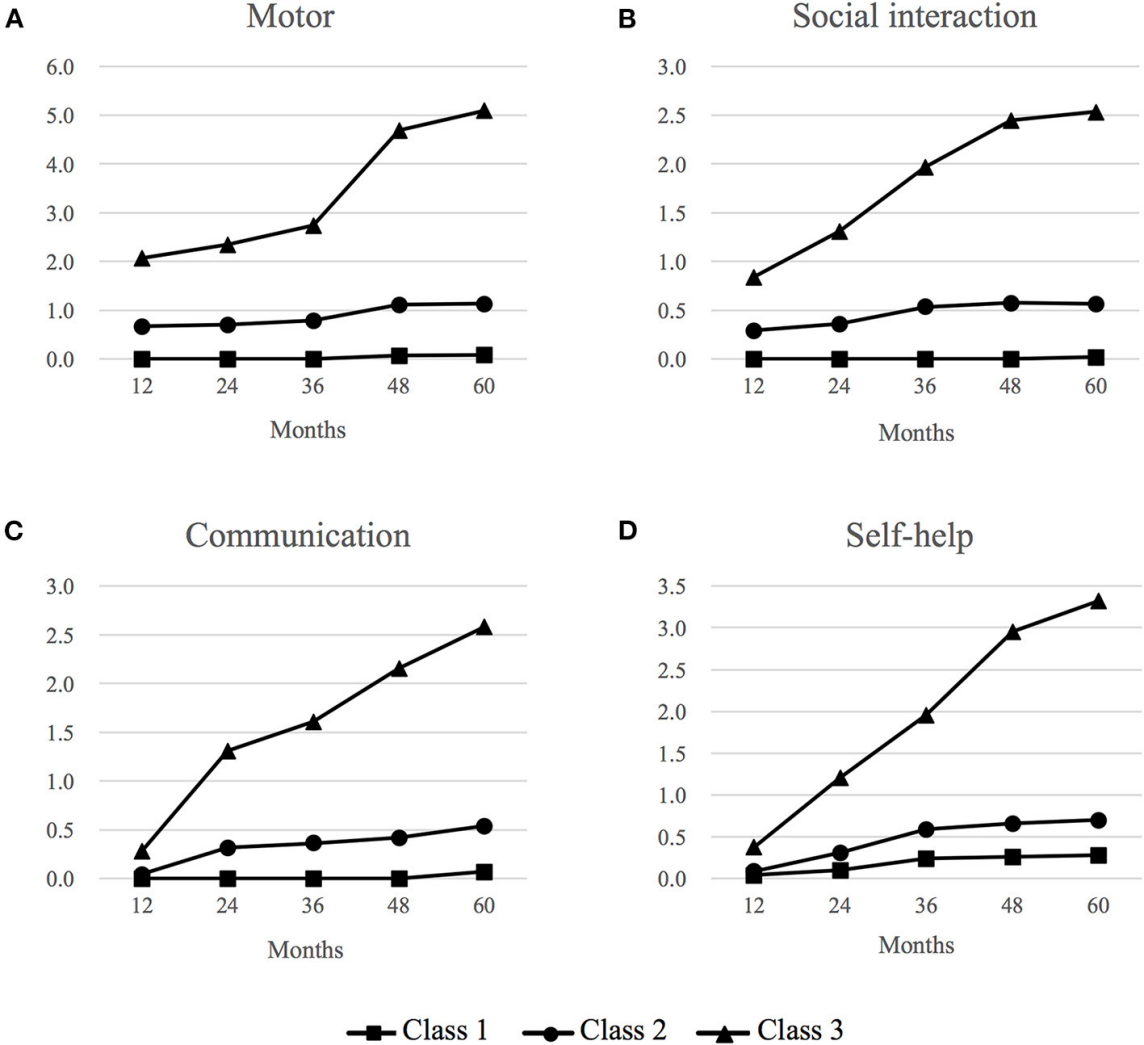
Trajectory patterns of milestone attainment in four developmental domains *via* parallel latent class growth analysis. **(A)** Motor domain, **(B)** Social interaction domain, **(C)** Communication domain, and **(D)** Self-help domain. Class 1 (42%): “Consistent attainment of expected milestones”, Class 2 (45%): “Initial slight delay and caught-up”, Class 3 (13%): “Consistent failure of expected milestone attainment”. Vertical values refer to the cumulative number of milestone items that were not attained at each expected time point. This was reported by the parents as “My child has not attained this developmental milestone”, indicating potential delays in milestone attainment.

We identified three distinctive trajectory patterns based on the observed values at each time-point per each milestone domain (Figure 2). Class 1 (42%) represents a group of children who consistently attained expected milestones throughout their early childhood up to 5 years of age (“Consistent milestone attainment” group). Class 2 (45%) was characterized with a group of children who did not attain a small number of milestones at the expected time range within the first 3 years of their life but caught up later on, represented by no further increases in the number of failed milestone attainment (“subtle initial delay and catch-up” group). The timing of the milestone non-attainment varied by each developmental domain; while non-attainment of a small number of milestones was observed by 12 months of age in the motor and social interaction domains, it occurred between 12 and 24 months in the communication domain and between 24 and 36 months in the self-help domain. Class 3 (13%) was characterized by consistent failure to attain expected milestones at each developmental time range, represented by the steady rise of the cumulative number of non-attainment of milestones over the 5 years (“Consistent failure to attain expected milestones” group).

The percentage of the NDD group and the non-NDD group per trajectory class are summarized in Table 3. In the group of children who continued to fail to attain expected milestones at each time range (Class 3, *N* = 90), the majority of children (90%, *n* = 81) received at least one NDD diagnosis. Focusing on this class, only 35 out of 81 children (43.2%) had a single NDD diagnosis, while the rest of 46 children (46.8%) had 2 or more co-occurring NDDs. The proportion of single NDD diagnosis and that of co-occurring NDDs, as well as distributional patterns of co-occurring NDDs, are summarized in Supplementary Material 4. Fifty-three out of 81 (65.4%) children were diagnosed with either ASD or ID or both, the proportion of which was higher than that in other two classes (31.4% in Class 1 and 42.3% in Class 2). In this study sample consisting of children who screened positive in the developmental screening, approximately half of the children who consistently attained expected milestones throughout early childhood (Class 1) received at least one NDD diagnosis.

**Table 3:** Percentage of NDD and non-NDD cases per trajectory class.

		No NDD*		**Any NDD**			
						**ASD and/or ID**	
	***N***	***n***	**%**	***n***	**%**	***n***	**% out of NDD**
Class 1	301	148	49.2	153	50.8	48	31.4
Class 2	313	105	33.5	208	66.5	88	42.3
Class 3	90	9	10.0	81	90.0	53	65.4

*ASD, autism spectrum disorder; ID, intellectual disability; NDD, neurodevelopmental disorder*.

*Class 1: “Consistent attainment of expected milestones.”*

*Class 2: “Initial slight delay and caught-up.”*

*Class 3: “Consistent failure of expected milestone attainment.”*

To clarify the influence of gestational age and sex on less favorable attainment patterns of developmental milestones, we chose Class 1 (“consistent milestone attainment”) as the reference class in multinomial logistic regression analyses (Table 4). Both male sex (Class 2 “initial slight delay and caught-up”: OR = 1.41, 95% CI: 1.02–1.95; Class 3 “consistent failure to attain expected milestones”: OR = 1.88, 95% CI: 1.13–3.13) and preterm birth (Class 2: OR = 1.95, 95% CI: 1.10–3.47; Class 3: OR = 3.53, 95% CI: 1.76–7.10) were associated with both classes, suggesting that children with male sex and preterm-born children were more likely to be assigned to Class 2 and Class 3 compared with Class 1.

**Table 4:** Predictors of less favorable trajectories of milestone attainment.

**Predictors**	**β**	**SE**	**Wald**	**df**	***p-*value**	**Exp (β)**	**95% confidence interval**
Class 2*							
Sex (male)	0.34	0.17	4.27	1	**0.24**	1.41	1.02–1.95
Preterm birth	0.67	0.30	5.07	1	**0.02**	1.95	1.09–3.47
Class 3*							
Sex (male)	0.63	0.26	5.91	1	**0.015**	1.88	1.13–3.13
Preterm birth	1.26	0.36	12.5	1	**<0.001**	3.53	1.76–7.10

^*^Class 1 as a reference in multinomial regression analysis.

*Class 1: “Consistent attainment of expected milestones.”*

*Class 2: “Initial slight delay and caught-up.”*

*Class 3: “Consistent failure of expected milestone attainment.”*

## Discussion

To our knowledge, this is the first study that elucidated trajectory patterns of attainment of developmental milestones during early childhood in a community sample of children at the risk of developmental disabilities in Japan. Using latent class growth analysis, we identified three different patterns of milestone attainment in a community sample of 720 children who presented to the comprehensive developmental assessment based on data charted on the MCHH from birth to 60 months of age. In addition to the two groups “consistent attainment of expected milestones” (42%) and “consistent failure to attain expected milestones” (13%), there was a group of children (45%) who did not attain a small number of milestones in one or more milestone domains within the first 36 months of life yet did not fail to attain expected milestones later on. This finding implies despite transient delays in milestone attainment during infancy and toddlerhood, some children can catch up with their peers. Our analysis did not reveal a subgroup of children who lost acquired skills (i.e., regression) and continued to fail to attain subsequent milestones. The low prevalence of etiologies that lead to developmental regression (epilepsy, for example) is likely a reason that we did not identify this pattern.

In the class where children consistently failed to attain expected milestones (Class 3), the majority of them were diagnosed with at least one NDD (81/90: 90%). Approximately 65% of children in Class 3 had ASD and/or ID, the rate of which was higher than that in the other two classes. The large proportion of these NDDs in Class 3 was consistent with our hypothesis made based on existing studies reporting atypical developmental trajectories in patients with these NDDs (13–15). Thus, this study finding indicates that MCHH can function as a useful tool in developmental surveillance and has potential utility in the early identification of these NDDs. However, it is important to note that while developmental surveillance *via* the MCHH can be useful by incorporating data *via* routine parental observation on the child's development and behaviors, it alone is not sufficient to capture who needs further evaluation (32). Inadequate accuracy with developmental surveillance alone is represented by our findings that a relatively high percentage of children with ASD alone, ID alone, or co-occurring ASD and ID in the other two classes. Lack of data on ASD severity and IQ in several children prohibit us from assessing how characteristics of children with these NDDs differ between Class 3 and other classes; however, we speculate Class 3 includes children with higher ASD severity and lower cognitive ability. Given the supplementary role of developmental surveillance in the early identification of certain NDDs, how combined MCHH data with commonly utilized screening tools for young children [M-CHAT, for example, (33)] can improve the accuracy of screening tools needs to be examined in future studies.

In all classes, approximately one-third of children received other NDDs, namely ADHD and/or DCD. Studies on early developmental signs or milestone attainment in these NDDs are scarce and findings could be biased due to the retrospective study designs (34–36). Additionally, these studies did not assess multiple developmental domains (only motor and language milestones for children later diagnosed with ADHD, for example). Our findings in four milestone domains (motor, social interaction, communication, and self-help) identified through the use of the MCHH data prospectively recorded by the parents can thus build a foundation of future research to elucidate early developmental pathways to these NDDs. While there is emerging evidence in early interventions for preschoolers with ADHD (37), early interventions for young children with DCD are not established (38) due to challenges with case ascertainment in early childhood. Therefore, furthering our understanding of what subgroups of children with developmental challenges in early childhood will eventually receive these NDD diagnoses can facilitate future studies examining the efficacy of certain early interventions commonly prescribed in pediatric practice (occupational therapy, physical therapy, for example).

As hypothesized, given male predominance in NDDs (39, 40), male sex predicted less favorable trajectory patterns of milestone attainment up to 5 years of age. Sex differences in milestone attainment in the present study may propose the necessity to develop sex-specific milestone charts. Taking ASD as an example of NDDs, studies have reported early skills development in girls (41) and later diagnosis of ASD in girls than in boys (42). Although multiple factors can contribute to it, delayed diagnosis of ASD in girls could be accounted for by currently available tools measuring milestone attainment as they may not capture both sexes equally. The same issue can apply to other NDDs ascertained in the present study given the male predominance of their estimated prevalence (38–40).

Consistent with our hypothesis, our findings revealed that preterm-born children were more likely to be assigned to less favorable trajectories of milestone attainment. Although parents are generally instructed to adjust their children's milestone attainment for gestational age in charting in the MCHH if their children are born prematurely, we did not confirm if they adjusted that way in the data entry process of the present study. Thus, caution is required in interpreting our findings. Findings from existing research are inconclusive regarding the most appropriate method for accounting for prematurity in developmental assessment (43), warranting the need for more studies.

Our findings on trajectory patterns of milestone attainment from birth to early childhood also have important implications for the use of the MCHH to improve children's health in global health research given the widespread use of the MCHH and similar HBRs in both high income and LMICs. Effective use of HBRs data can lead to early detections of the child's developmental delays and challenges and subsequently can improve community-based primary health care, satisfying one of the Sustainable Development Goals marked by the World Health Organization (44). However, poor access to early interventions in resource-constrained settings and countries would remain a challenge in achieving this goal (45). To this aim, further research is required to develop approaches allowing data obtained from HBRs, including the MCHH, to be integrated into existing resources and health systems and to inform actions for early interventions.

Our study has the following limitations. First, language and speech disorders, which are important NDDs, were not assessed using standardized assessment tools at 5 years of age. Second, we did not examine the effect of early intervention on the child's milestone attainment. This could be achieved in future larger sample-sized studies with information on the modality, timing (at what age the intervention is started), and duration of the early interventions. Third, although ascertained through an epidemiological survey conducted in a total population sample, participants in the present study were children whose parents reported certain developmental concerns and who underwent the in-person developmental assessment. Thus, we are unable to extrapolate our findings to a general population sample. Given the nature of the MCHH, designed as a tool under the universal healthcare system, future studies need to assess the utility of the MCHH in a total population sample. Lastly, modest sample size and ethnically and culturally homogeneous participants in the present study can be the study limitations.

Despite the limitations above, our present study has notable strengths in the study design. In the present study, we assumed that most parents prospectively charted their children's milestone attainment at each time point in the MCHH as they were educated to bring the MCHH to well-being checkups. Therefore, we believe findings in the present study were minimally affected by recall bias. The other major strength was the thoroughness of case ascertainment of NDDs *via* in-person diagnostic assessment by multidisciplinary professionals, making diagnoses more accurate than other studies where NDDs were diagnosed without in-person assessment [through national registry data, for example: (13)].

## Conclusion

Using milestone data obtained *via* the MCHH, we identified three different trajectory patterns of milestone attainment up to 5 years of age. In the class where children consistently failed to attain expected milestones in motor, social interaction, communication, and self-help domains, the majority of them received at least one NDD diagnosis through comprehensive evaluation at age 5, implying the potential utility of the MCHH as a tool providing parents with guidance on what patterns of milestone attainment they need to be concerned about and when to bring their concerns to pediatricians. Although the use of the MCHH alone does not substitute diagnostic evaluation, parental concerns raised based on routine monitoring of the child's development through the MCHH and certain aberrant patterns of milestone attainment may lead to early intervention that can target broad developmental areas. From this perspective, the MCHH can serve as an effective tool in developmental surveillance. Combining the MCHH data with existing developmental screening tools may improve accuracy of early detections of NDDs.

## Data Availability Statement

The raw data supporting the conclusions of this article will be made available by the authors, without undue reservation.

## Ethics Statement

The studies involving human participants were reviewed and approved by the Committee of Medical Ethics of Hirosaki University Graduate School of Medicine. Written informed consent to participate in this study was provided by the participants' legal guardian/next of kin.

## Author Contributions

TH, TN, MS, and KN contributed to the conception and design of the study. TH wrote the first draft of the manuscript. TH and MS collected the data and organized the database. TH, TN, and MM performed the statistical analysis. TN, MM, MS, and KN critically reviewed the manuscript. All authors contributed to manuscript revision, read, and approved the submitted version.

## Funding

This research was financially supported by the Hirosaki Institute of Neuroscience in Japan (KN), Hirosaki University Institutional Research Grant (KN), Hirosaki University Institutional Research Grant for Future Innovation (MS), and Japan Society for the Promotion of Science (JSPS) KAKENHI, Grant Numbers: 19K08008 (TH), 21K20256 (MM), and 20H03595 (KN). Contract research project from Hirosaki City (MS).

## Conflict of Interest

The authors declare that the research was conducted in the absence of any commercial or financial relationships that could be construed as a potential conflict of interest.

## Publisher's Note

All claims expressed in this article are solely those of the authors and do not necessarily represent those of their affiliated organizations, or those of the publisher, the editors and the reviewers. Any product that may be evaluated in this article, or claim that may be made by its manufacturer, is not guaranteed or endorsed by the publisher.
