# Supplementary material for: The Role of the Maternal and Child Health Handbook in Developmental Surveillance: The Exploration of Milestone Attainment Trajectories
Source: Front Psychiatry. 2022 Jun 17;13:902158. doi: 10.3389/fpsyt.2022.902158 (PMC9247330; doi:10.3389/fpsyt.2022.902158)
Supplement: Supplementary file 1 [file Table_1.DOCX]

Supplementary Material

**Supplementary Material 1. Diagnostic testing and case ascertainment**

For children who attended the diagnostic assessment, we conducted the Diagnostic Interview for Social and Communication Disorders (DISCO) (1), a semi-structured interview designed to obtain information on the child’s development and behaviors and can be used to assist diagnoses of autism spectrum disorder (ASD) and related neurodevelopmental disorders. A child and adolescent psychiatrist (MS) and graduate students supervised the child psychiatrists conducted the DISCO.

All children underwent non-structured interview and behavior observation, conducted by the board-certified child and adolescent psychiatrists. All interviews and behavior observations were videotaped and reviewed by the research team.

Cognitive assessment was conducted only in children who had not received a diagnosis of intellectual disability (ID) prior to this diagnostic assessment. For those children, the Japanese version of the Wechsler Intelligence Scale for Children, 4th edition (WISC-IV) (2) was conducted by a psychologist. For children with a previously established ID diagnosis, we examined the disability certificate as a reference.

Additionally, children underwent motor skills and coordination assessment via the Movement Assessment Battery for Children, 2nd edition (MABC-2), a tool consisting of eight tasks: three measure manual dexterity, two-measure ball skills, and three measure balance (3), conducted by licensed occupational therapists and psychologist.

Each case was reviewed and discussed in a multidisciplinary team comprising psychiatrists, psychologists, a pediatrician, and occupational therapists. These multidisciplinary professionals produced the best-estimate diagnoses based on the Diagnostic and Statistical Manual of Mental Disorders, Fifth Edition.

**Supplementary Material 2. Missing data per each developmental domain at each time range**

| Age | Domain | Missing data (%) |
| --- | --- | --- |
| 12 months or earlier | Motor | 7.4 |
|  | Social interaction | 8.4 |
|  | Communication | 10.1 |
|  | Self-help | 8.6 |
| 24 months or earlier | Motor | 5.8 |
|  | Social interaction | 6.6 |
|  | Communication | 6.7 |
|  | Self-help | 6.3 |
| 36 months or earlier | Motor | 5.3 |
|  | Social interaction | 5.9 |
|  | Communication | 6.1 |
|  | Self-help | 5.8 |
| 48 months or earlier | Motor | 41.7 |
|  | Social interaction | 34.4 |
|  | Communication | 35.4 |
|  | Self-help | 35.1 |
| 60 months or earlier | Motor | 45.5 |
|  | Social interaction | 40.1 |
|  | Communication | 41.3 |
|  | Self-help | 37.9 |

**Supplementary Material 3. Class determination processes in parallel latent class growth analysis**

Although the Bayesian information criterion (BIC) value was lower in a four-class solution and a five-class solution than in a three-class solution, the *p*-value of the likelihood ratio test (VLMR-LRT) assessing the goodness of fit of two competing statistical models based on the ratio of their likelihoods, became nonsignificant in the four-class solution. Additionally, careful examination of trajectory patterns identified in the four-class solution revealed that the two of them were not conceptually different. Therefore, we determined the three-class solution as the optimal model.

**Supplementary Material 4. Patterns of co-occurring neurodevelopmental disorders in Class 3 “Consistent failure of milestone attainment” (N = 81)**

|  | n | % |
| --- | --- | --- |
| ASD alone | 5 | 6.2 |
| ADHD alone | 5 | 6.2 |
| DCD alone | 21 | 25.9 |
| ID alone | 4 | 4.9 |
| ASD + ADHD | 1 | 1.2 |
| ASD + DCD | 2 | 2.5 |
| ASD + ID | 5 | 6.2 |
| ADHD +DCD | 2 | 2.5 |
| ADHD + ID | 0 | 0 |
| DCD + ID | 12 | 14.8 |
| ASD + ADHD + DCD | 4 | 4.9 |
| ASD + ADHD + ID | 1 | 1.2 |
| ASD + DCD + ID | 9 | 11.1 |
| ADHD + DCD + ID | 0 | 0 |
| ASD + ADHD + DCD + ID | 10 | 12.4 |
| Total | 81 | 100 |

Single NDD (n = 35, 43.2%), Co-occurring NDDs (n = 46, 56.8%): two NDDs (n = 22); three NDDs (n = 14); four NDDs (n = 10)

ASD = autism spectrum disorder, ADHD = attention-deficit hyperactivity disorder, DCD = developmental coordination disorder, ID = intellectual disability, NDD = neurodevelopmental disorder

**Reference**

1. Wing L, Leekam SR, Libby SJ, Gould J, Larcombe M. The Diagnostic Interview for Social and Communication Disorders: background, inter-rater reliability and clinical use. J Child Psychol Psychiatry. 2002 Mar;43(3):307–25.

2. Wechsler D. Wechsler intelligence scale for children (4th ed.). San Antonio, TX: Psychological Corporation; 2003.

3. Henderson S, Sugden D, Barnett A. The movement assessment battery for children. 2nd ed. London: The Psychological Corporation; 2007.
